# Bayesian Mediation Analysis with an Application to Explore Racial Disparities in the Diagnostic Age of Breast Cancer

**DOI:** 10.3390/stats7020022

**Published:** 2024-04-19

**Authors:** Wentao Cao, Joseph Hagan, Qingzhao Yu

**Affiliations:** 1 Louisiana Department of Education, 1201 N 3rd St, Baton Rouge, LA 70802, USA; 2 Department of Pediatrics, Baylor College of Medicine, 1 Baylor Plz, Houston, TX 77030, USA; 3 School of Public Health, Louisiana State University Health–New Orleans, 3rd Floor, 2020 Graviers Street, New Orleans, LA 70112, USA

**Keywords:** mediation effect, Bayesian mediation analysis methods, breast cancer

## Abstract

A mediation effect refers to the effect transmitted by a mediator intervening in the relationship between an exposure variable and a response variable. Mediation analysis is widely used to identify significant mediators and to make inferences on their effects. The Bayesian method allows researchers to incorporate prior information from previous knowledge into the analysis, deal with the hierarchical structure of variables, and estimate the quantities of interest from the posterior distributions. This paper proposes three Bayesian mediation analysis methods to make inferences on mediation effects. Our proposed methods are the following: (1) the function of coefficients method; (2) the product of partial difference method; and (3) the re-sampling method. We apply these three methods to explore racial disparities in the diagnostic age of breast cancer patients in Louisiana. We found that African American (AA) patients are diagnosed at an average of 4.37 years younger compared with Caucasian (CA) patients (57.40 versus 61.77, *p* < 0.0001). We also found that the racial disparity can be explained by patients’ insurance (12.90%), marital status (17.17%), cancer stage (3.27%), and residential environmental factors, including the percent of the population under age 18 (3.07%) and the environmental factor of intersection density (9.02%).

## 1. Background

Mediation analysis is widely used to identify significant variables (called mediators) and estimate their effects in pathways between an exposure variable and a response variable. It is broadly used in many fields, such as social science, psychology, and epidemiology. In the application example, we found that African Americans were diagnosed with breast cancer at an average younger age compared with Caucasian American (CA) women. We are interested in investigating risk factors that can explain the racial disparity in the diagnostic age of breast cancer patients. In the example, the exposure variable is race, the outcome is the diagnostic age, and all variables potentially explaining the racial disparity are mediators. In mediation analysis, the indirect effect of a mediator refers to the effect transmitted by a mediator intervening in the relationship between an exposure variable and a response variable. The direct effect refers to the effect between the exposure and outcome variables after adjusting for all mediators. To estimate the mediation effects, there are three conventional methods: (1) the difference in the coefficients method; (2) the product of the coefficients method; and (3) the counterfactual framework method [1–6].

Based on the counterfactual framework method, Yu et al. generalized the concept of mediation effects and developed a general multiple mediation analysis method (referred to as the Yu method [7]). To illustrate the method, we denote the exposure variable as X, the response variable as Y, and the mediator as M. By the definition of the Yu method, the total effect of X on Y at $X=\;x^{*}$ is defined as the changing rate in E(Y(X)) when X changes: $TE\left(x^{*}\right)={lim}_{u\to u^{*}}\;\frac{E\left[Y\left(x^{*}+u\right)-Y\left(x^{*}\right)\right]}{u}$, where $u^{*}$ is an infimum changing amount in X. The direct effect of X at $x^{*}$ is defined as $DE_{m}=E_{m}[{lim}_{u\to u^{*}}\;\frac{E\left(Y\left(x^{*}+u,M=m\right)-E\left(Y\left(x^{*},M=m\right)\right.\right.}{u}].$ The indirect effect of X on Y through M is the difference between the total and direct effects. Compared with the conventional definition of mediation effects, where the average mediation effects are defined as the average change amount in the potential outcome when the exposure variable is at different levels [2,8,9], the definition of mediation effects in the Yu method is based on the rate of change. By definition, the mediation effects are generalized so as to deal with different formats (e.g., binary, multi-categorical, and/or continuous) of exposure variables, mediators, and response variables. Yu et al. further extend the method to be used with different predictive models [10].

Unlike the frequentist method, the Bayesian approach assumes that the parameters of interest are random rather than fixed variables. Prior distributions are assigned, and the quantities of interest are inferred from the posterior distributions. The Bayesian method, in general, has many benefits. First, it allows the researchers to incorporate previous knowledge and information from the data set into the analysis. Incorporating accurate prior information into the mediation analysis can improve the efficiency of estimates. Second, the inferences are based on posterior distributions. Therefore, the interpretation of inferences is straightforward. For example, unlike the 95% confidence interval obtained using the frequentist method, a 95% credible set built by the Bayesian method can be interpreted as the parameter being in the credible set with a 95% probability.

There are numerous advantages to utilizing the Bayesian method in mediation analysis. Firstly, it allows us to make inferences on mediation effects based on the posterior distributions of parameters, eliminating the need for bootstrap sampling as we can directly obtain variances of estimates. Secondly, parameters are considered random variables rather than fixed values in the Bayesian hierarchical structure. This makes it natural to assign distributions to mediators that are influenced by exposure variables and covariates. Lastly, the Bayesian framework inherently incorporates mediation analysis with multilevel models, further enhancing its applicability.

Several Bayesian mediation analysis approaches have been published. The earliest method, introduced by Yuan et al. in 2009 [11], employed Bayesian regressions to model variable associations and utilized the coefficient product (CP) method for estimating indirect effects. Yuan et al. discovered that Bayesian methods outperformed frequentist methods, particularly in scenarios with small to moderate sample sizes. Subsequent research by Miočević et al. [12,13] further evaluated the method’s efficacy across varying sample sizes. However, like the CP method it inherits, their Bayesian approach is primarily suited for continuous mediators and does not readily extend to other types, such as binary or categorical mediators. Later advancements by Park [14] and Kim [15] introduced Bayesian mediation analysis grounded in the counterfactual framework, where indirect effects are computed as the difference in potential outcomes between two levels of the exposure variable. These methods demonstrated efficiency through both simulation studies and real-world examples. Similarly, techniques for multilevel mediation analysis were developed to investigate risk factors within hierarchical data structures [16,17]. While these potential outcome methods can accommodate nonlinear relationships among variables, they are constrained to binary exposure variables.

In this paper, we present and compare three Bayesian mediation analysis methods. Our methods’ contribution is that we applied the general definitions of the mediation effects in the Bayesian setting. Therefore, our methods can deal with nonlinear relationships among variables and different types of mediators, exposures, and outcomes. The methods are applied with the Louisiana Tumor Registry data to investigate racial disparities observed in the breast cancer onset age. The rest of the paper is organized as follows. In Section 2, we introduce a data set that is used to explore racial disparity in breast cancer diagnostic ages. Then, in Section 3, we review three different Bayesian methods proposed by Yu et al. [18] to estimate the mediation effects for continuous/binary exposure variables with multivariate outcomes. We apply these methods to explore the racial disparity in the diagnostic age of breast cancer patients in Louisiana, and the results are presented in Section 4. In Section 5, we present the conclusions and point out future research directions.

## 2. Data Set and Variable Description

According to statistics from the Centers for Disease Control and Prevention, breast cancer has become the second most common cancer in women in the United States. It has been observed that African American (AA) women have a higher risk of dying of breast cancer compared with Caucasian (CA) women. However, AA women have a lower breast cancer incidence [19–21]. Previous studies proposed several potential risk factors that may contribute to the racial disparity in breast cancer, such as residential environment risk factors, treatments, individual behaviors, and tumor subtypes [22–24]. As an initial study, Yu et al. found, through a general mediation method, that being diagnosed at an early age, being diagnosed at an early stage, being estrogen receptor (ER)–/progesterone receptor (PR)-positive, having a low tumor grade, having surgery, and receiving hormonal therapy were related to a better survival rate among breast cancer patients [7]. They also found that, on average, AA women were more likely to be diagnosed at younger ages compared to CA women. The purpose of this study is to identify which factors could be important in explaining the racial disparity in the diagnostic age of breast cancer from a large pool of potential risk factors. The research result provides information on the potential for delaying the incidence age of breast cancer by designing interventions that may control the related factors. We propose a Bayesian mediation analysis method for the study data to differentiate the effects from various risk factors.

The data set used in this study is from the comparative effectiveness research patient-centered outcomes research (CER-PCOR), supported by the Centers for Disease Control and Prevention (CDC). The CER-PCOR is collecting longitudinal follow-up data from 2011 diagnosed cancer cases, such as breast and colon cancer. The CER-PCOR data set includes the North American Association of Central Cancer Registries’ (NAACCR) standardized variables on patient and tumor characteristics and patient follow-up, variables on the first course of cancer-related treatment, and disease progression and recurrence status. The project includes all non-Hispanic white and black women diagnosed with invasive breast cancer in Louisiana in 2011. In this research, to investigate the racial disparity in breast cancer ages, we include both patient characteristics and their residential environmental variables. Specifically, variables on patient characteristics include age at diagnosis, race, marital status, insurance, body mass index, comorbidity, molecular cancer type, income, and education. The census tract-level contextual variables include intersection density, street density, connection node ratio, language isolation, and the percent of the population under the age of 18. The census-tract level variables (neighborhood environment data) are linked to the Louisiana Tumor Registry database through patients’ geographic identifiers.

The neighborhood environment data are generated based on the 2010 Census and the American Community Survey. The variable intersection density is measured as the number of intersections per square mile of area. Street density is calculated by the number of linear miles of street per square mile of area. The connected node ratio refers to the ratio of the number of intersections with four or more connections over the total number of intersections. We use the three variables to measure the overall construct of neighborhood walkability [25]. The overall neighborhood walkability is considered a surrogate factor associated with physical activity [26]. Some previous studies show that increased physical activity is related to a reduced risk of breast cancer and a prolonged lifetime after developing breast cancer [27–29]. In our study, we explore the potential effect of walkability in explaining the diagnostic age of breast cancer.

In total, there were 2275 non-Hispanic white (1579) and black female patients (696) who were diagnosed with invasive breast cancer. All cases identified from death certificates only were excluded. We found that the average diagnostic age of breast cancer for black women was significantly younger than that for white patients (57.40 versus 61.77, *p* < 0.0001). The purpose of this research is to investigate risk factors that may explain the racial disparity in the diagnostic age of breast cancer patients. The binary exposure variable is race, and the continuous outcome is the diagnostic age of breast cancer. All potential mediators and their summary statistics are listed in Table 1.

In Table 1, the descriptive statistics for each variable are shown by the AA and CA patients separately. Compared with white patients, black patients are more likely to be diagnosed with advanced cancer stages and have a higher BMI and a higher poverty index. A variable is identified as a potential mediator for racial disparity if two conditions are satisfied [30]. First, the variable has to be distributed differently between the AA and CA patient populations. To test this, we use the chi-square test for categorical variables and the analysis of variance (ANOVA) test for continuous variables. The *p*-values for these tests are listed as *p*-value 2 in Table 1. The second condition is that the variable is significantly related to the outcome, adjusting for other factors potentially related to the outcome. The second condition is tested through generalized linear models, and results of these tests are listed as *p*-value 1 in Table 1. If both conditions are satisfied (both *p*-values are smaller than 0.1), we include the variable in further analysis as a mediator. Note that the two tests are used as pre-screening but not as formal tests for mediation. Variables that are considered critical in explaining the racial disparity from the literature or hypotheses can be forced into the analysis as potential mediators without the tests of the two conditions. In this study, intersection density, street density, and connection node ratio (overall construct of neighborhood walkability) are forced into the final model, and we are interested in finding their joint effect on racial disparity.

## 3. Methods

In this section, we briefly review the three Bayesian methods for mediation analysis [18]. The first method is based on the functions of estimated parameters, the second method is an extension of the product of coefficients method, and the third method is a counterfactual resampling method. For simplicity, we assume there is only one exposure variable X, one outcome Y, and one potential third variable M. Detailed algorithms are provided in the Appendix of [18].

### 3.1. Method 1: Functions of Estimated Coefficients

Under certain modeling and assumptions, mediation effects can be estimated directly as analytical functions of the coefficients from fitted models. For this method, we first compute mediation effects based on the predictive models for relationships among variables. The mediation effects are then calculated using posterior distributions of parameters for the predictive models. The method is typically used when generalized linear models are used to fit relationships among variables. For example, let a single-level mediation model for the outcome have the following format:

(1) $$Y\;=\;i_{0}\;+\;cX\;+\;bM\;+\;e_{1}$$

(2) $$M\;=\;i_{1}\;+\;aX\;+\;e_{2}$$

Under the Bayesian analysis framework, prior distributions are assigned to the coefficients and other parameters. By definition, the indirect effect of M is defined as the effect of X on Y through M. Samples of a and b are drawn from the posterior distributions using Gibb’s sampling. Using the *ith* paired sample, denoted as $\left({\hat{a}}_{i},\;{\hat{b}}_{i}\right)$, drawn from posterior distributions, a sample of the indirect effect drawn from its posterior distribution is ${\hat{a}}_{i}\;\times \;{\hat{b}}_{i}$. Similarly, if the outcome Y is binary and fitted by the logit model,

(3) $$log\frac{P(Y\;=\;1)}{P(Y\;=\;0)}=\;i_{2}+c_{1}X+b_{1}M.$$

Samples a and $b_{1}$ are drawn from their posterior distributions, and the samples with an indirect effect from M can be calculated by $a\times b_{1}\times \frac{exp\left(i_{2}+c_{1}X+b_{1}M+e_{3}\right)}{{\left(1+exp\left(i_{2}+c_{1}X+b_{1}M+e_{3}\right)\right)}^{2}}$, replacing the parameters with the posterior samples for related parameters. The direct effect can be calculated by $c_{1}\times \frac{exp\left(i_{2}+c_{1}X+b_{1}M+e_{3}\right)}{{\left(1+exp\left(i_{2}+c_{1}X+b_{1}M+e_{3}\right)\right)}^{2}}$. Readers are referred to [7] for the calculations. The benefit of Method 1 is that the computation of the mediation effect is straightforward. However, when different models are assumed, it might be difficult to derive the analytical functions of the coefficients to estimate the mediation effects.

### 3.2. Method 2: Mediation Effects as the Product of Partial Differences

Method 2 estimates the mediation effects through the partial difference. To illustrate this method, we denote the estimation function of the exposure variable X on the mediator M as E(M)=g(M|X) and the estimation function of the outcome Y as E(Y)=f(Y|X,M). If f(Y|X,M) is differentiable with respect to X and M, the direct effect of the exposure variable on the outcome can be calculated by $DE(x)={|\frac{\partial f(Y|X,M)}{\partial X}}_{X=x}$. If g(M∣X) is differentiable based on X, the indirect effect from X to Y through M can be calculated as $IE_{M}(x)={\frac{\partial g(M|X)}{\partial X}\cdot \frac{\partial f(Y|X,M)}{\partial M}|}_{X=x}$, where the first part, $\frac{\partial g(M|X)}{\partial X}$, measures the partial changes in M when X changes, and the second part, ${\frac{\partial f(Y|X,M)}{\partial M}|}_{X=x}$, is the partial change in Y when M changes. To make the method more general without the need to take derivatives of different models, we replace the differentiation with the differences, such that DE(x) is calculated as

(4) $$\frac{f(Y|X\;=\;x\;+\;\Delta x,\;M(x))\;-\;f(Y|X\;=\;x,\;M(x))}{\Delta x}$$

and ${IE}_{M}(x)$ is calculated as

(5) $$\frac{g(M|X=x+\Delta x)-g(M|X=x)}{\Delta x}\cdot \frac{f(Y|X=x,M(x)+\Delta m)-f(Y|X=x,M(x))}{\Delta m}$$

To obtain accurate inferences, we choose Δx and Δm to be small, for example, the minimum of 0.01 or the range of X or M divided by 100.

Under the Bayesian analysis framework, samples are drawn from the posterior predictive distributions for f(Y|X,M) and g(M|X), and then the indirect effects of M are calculated using Equation (5). The benefit of using Method 2 is that mediation effects can be calculated without knowing the transformation functions.

### 3.3. Method 3: Re-Sampling Method

Method 3 is analogous to the re-sampling method in the Yu method. The total effect is defined as the changing rate in the outcome when the exposure variable changes, along with changes in all mediators, and it is calculated as $\frac{f(x+\Delta x,M(x+\Delta x))-f(x,M(x))}{\Delta x}$. The direct effect not from M is calculated as $\frac{f(x+\Delta x,M)-f(x,M)}{\Delta x}$, where M is drawn from its marginal distribution. The indirect effect of X on Y through M is then the difference between the total effect and the direct effect of X on Y:

(6) $$\frac{\left[f(Y|X=x+\Delta x,M(x+\Delta x)-f(Y|X=x,M(x))]-[f(Y|X=x+\Delta x,M)-f(Y|X=x,M)\right]}{\Delta x}.$$

As in Method 3, we choose Δx as the minimum of 0.01 or the range of X divided by 100. When Δx is very small, the number of Markov chain Monte Carlo (MCMC) iterations should be large enough to ensure the convergence of the estimation results. Under the Bayesian analysis framework, samples are drawn from the posterior predictive distributions for f(Y|M,X) conditional on different sets of exposures and mediators. Then, the indirect effect of M is calculated using Equation (6). Method 3 is the most general method and can be used with any predictive model.

### 3.4. Prior Distribution and Computational Method

In general, prior distributions for parameters are set up by choosing means reflecting prior knowledge and variances representing uncertainties. For example, a large variance is assigned in a prior distribution if there is not much prior information about a parameter. Typical prior distributions for coefficients in Bayesian regressions can be used in the Bayesian mediation analysis. For example, prior distributions for coefficients are generally normal, Laplace, or non-informative. The error terms are generally assigned with gamma prior distributions for computational convenience since they are conjugate priors [31].

MCMC methods, such as the Gibbs sampler and the Metropolis–Hastings algorithm, are widely used to draw samples from the posterior distribution of quantities of interest. By increasing the number of iterations, the samples obtained using MCMC methods gradually converge to the target distribution. In this paper, we perform all the analyses using JAGS: a Bayesian program for hierarchical models using Gibbs sampling [32]. The codes for the Bayesian mediation and the JAGS model used are presented in the Supplement Material, Sections S1 and S2.

## 4. Results

We used all three of the Bayesian methods by Yu et al. [18] with the study data to explore racial disparity in the diagnostic ages of breast cancer. Each mediator was fit with the predictor race using a Bayesian generalized linear model. We assumed that all coefficients have an independent prior normal distribution with mean 0 and the precision term 1.0 × 10^−6^. We also assigned all variance terms to an independent prior gamma distribution with a shape of 1 and a rate of 0.1. We performed one chain of MCMC sampling from the posterior distributions; 11,000 samples were drawn from the posterior distributions, and the first 1000 iterations were thrown away (burn-ins). We checked and confirmed the convergence of the posterior distributions by looking at the traces and the autocorrelation coefficient plots. If there were no computational constraints, the multiple chains traversing the same parameter space also justified the convergence. Since Bayesian generalized linear models with non-informative priors were used in the project, the results of these three methods turned out to be very close to each other. We show the results from Method 3 only. Table 2 shows the estimated mediation effects with their 95% credible sets.

The total effect is −4.37 with a 95% credible set (−5.47, −3.26), which means that, compared with CA patients, AA patients were diagnosed at an average age of 4.37 years younger. Since the credible set does not include 0, AA women were diagnosed at a significantly younger age than CA women. The indirect effect of a mediator can be negative or positive. If the indirect effect is negative, the same sign as the total effect, it means that the corresponding risk factor helps to explain part of the racial disparity. Otherwise, a positive effect means that, adjusting for the mediator, the racial disparity is going to be enlarged rather than explained. After adjusting for all mediators, the direct effect is −4.88, which is even smaller than the total effect. This means that the racial disparity would become larger if all mediators could be adjusted to be distributed evenly among the AA and CA patient populations. The positive effects are mainly from the isolation, poverty, and walkability factors.

**Figure 1.**
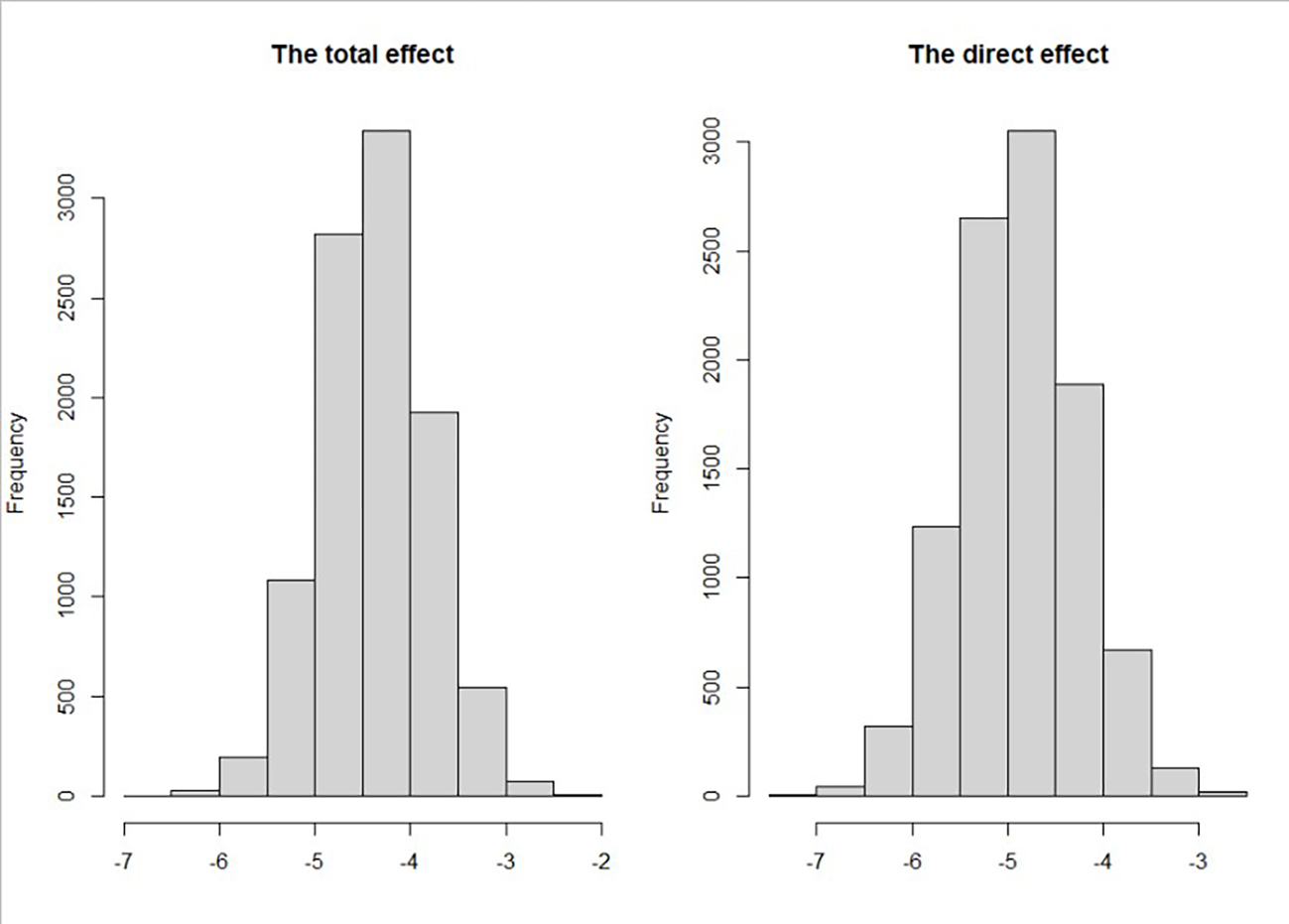
Posterior distribution of diagnostic age differences (AA–CA) for the total effect and direct effect.

**Figure 2.**
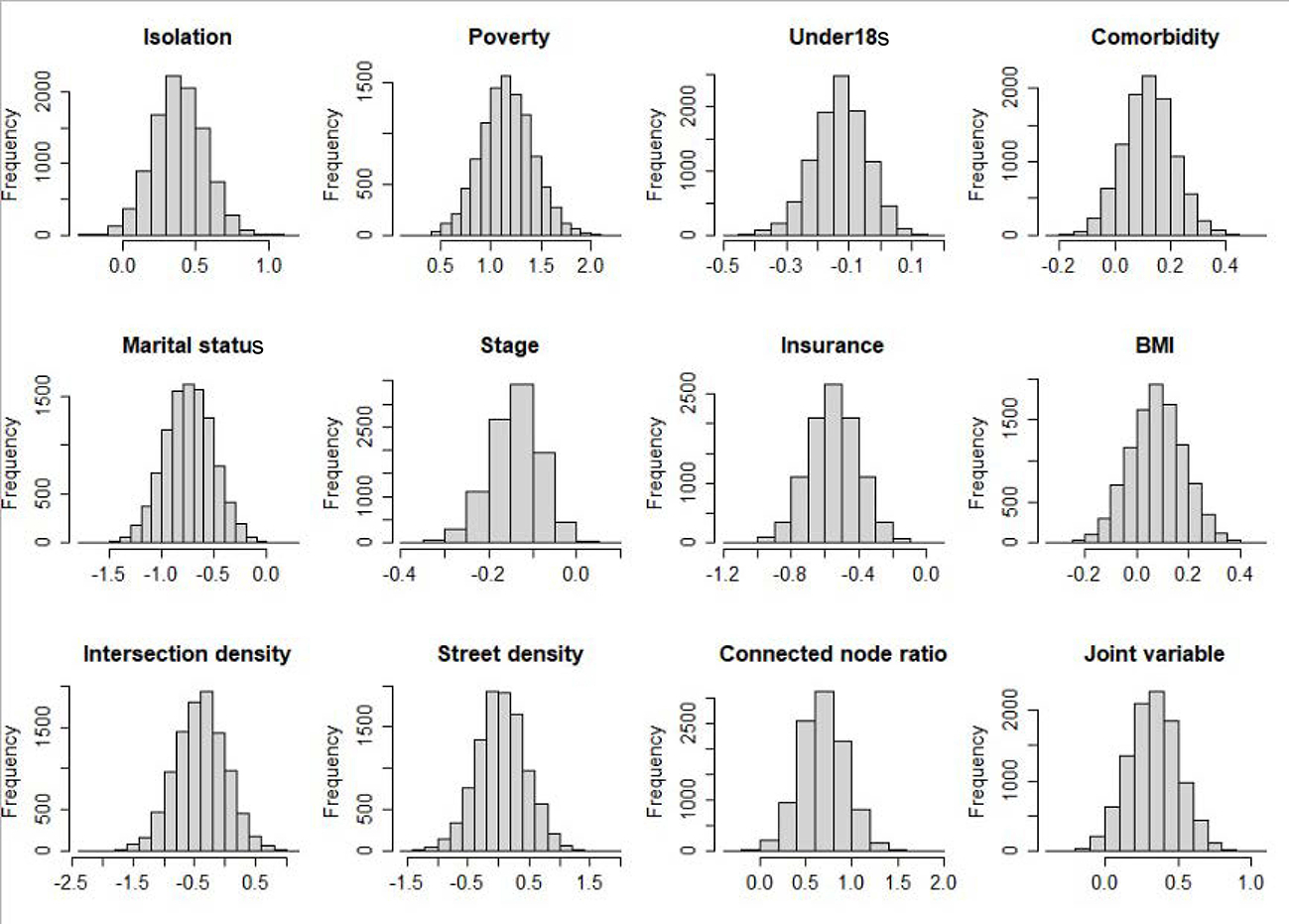
Posterior distribution for the indirect effect of each mediator in explaining the racial/ethnic disparity in the diagnostic age of breast cancer.

The posterior distributions for the total effect and the direct effect are shown in Figure 1. We found that the posterior distributions for both the total effect and direct effect are perfectly symmetric and are to the left of 0. Compared with white patients, black patients have an average younger diagnostic age, whether adjusting for mediators or not. Figure 2 shows the posterior distributions of indirect effects for all mediators. We found that the posterior distributions of indirect effects are not necessarily normal.

**Figure 3.**
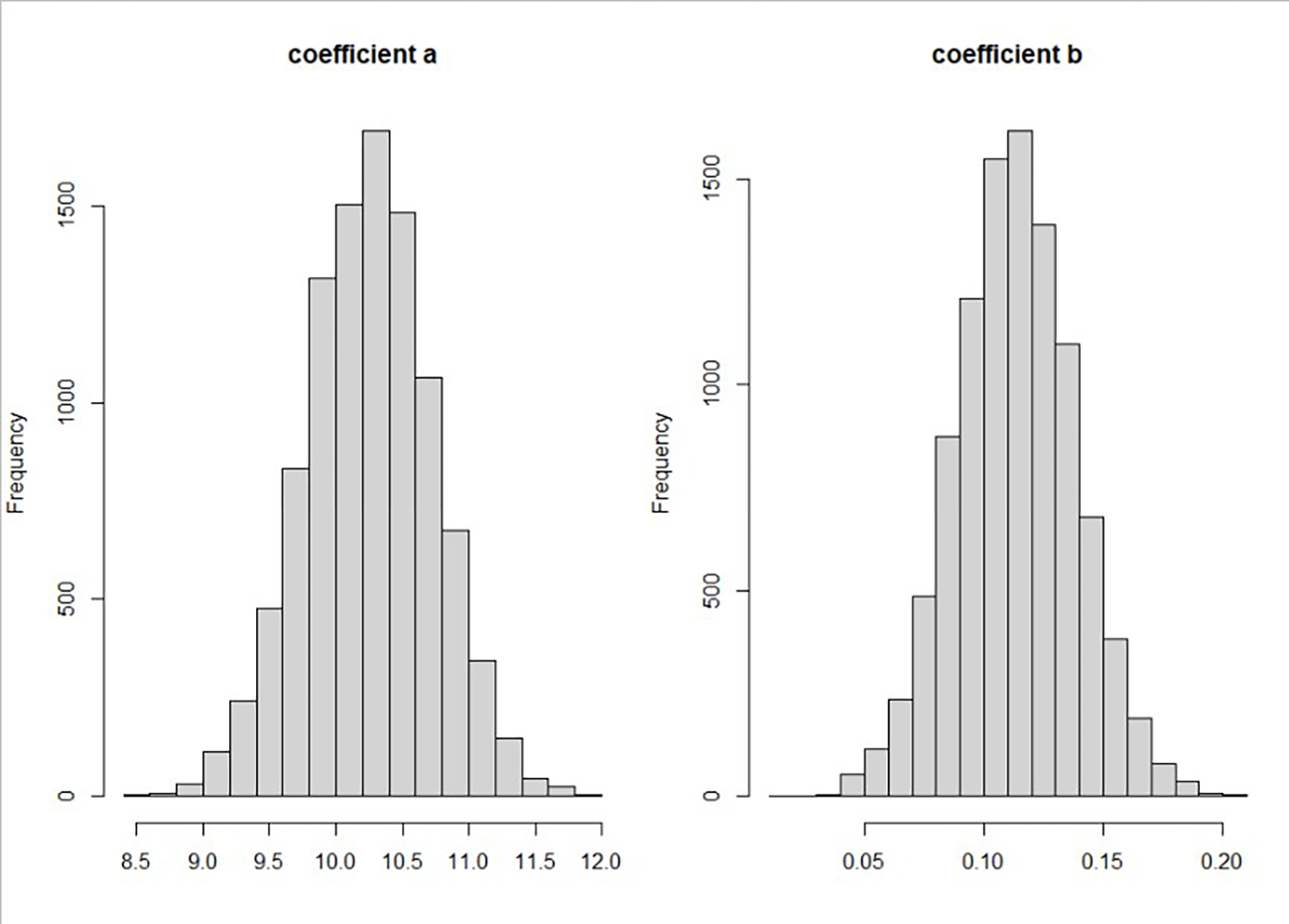
Posterior distribution for coefficients relating to poverty. The posterior distribution of the coefficient a for race to predict poverty index (**left panel**) and the posterior distribution of the coefficient b for poverty index to predict the diagnostic age of breast cancer (**right panel**).

The posterior distribution for the indirect effect of the poverty index is to the right of 0. The left panel of Figure 3 shows the posterior distribution of the coefficient for race when it is used to predict the poverty index, and the right panel shows the posterior distribution of the coefficient for poverty index when it is used to predict the diagnostic age of breast cancer with other variables. The posterior distribution of the coefficient of race is dominantly to the right of 0, meaning that black (race/ethnicity = 1) patients generally had a higher poverty index than white patients. The posterior distribution of the coefficient for the poverty index is also dominantly to the right of 0, which means that a higher poverty index is related to an older diagnostic age of breast cancer. A potential explanation is that wealthier patients are more likely to have cancer screenings, such as mammograms. Screening could help diagnose breast cancer early. This may also explain why poor patients are more likely to be diagnosed at advanced cancer stages.

When a mediator was multi-categorical with K categories, we transformed the variable into K-1 binary variables. Then, the joint effects of the transformed variables were calculated. For example, the variable insurance has three levels: (1) no insurance, (2) public insurance, and (3) private insurance. We used the first level, no insurance, as the reference level. The relative probability of each level depends on the exposure variable X, which was fit by

(7) $$log\frac{P(\text{Public-insurance})}{P(\text{No-insurance})}=a_{01}+a_{1}\times X_{i}$$

(8) $$log\frac{P(\text{Private-insurance})}{P(\text{No-insurance})}=a_{02}+a_{2}\times X_{i}$$

**Figure 4.**
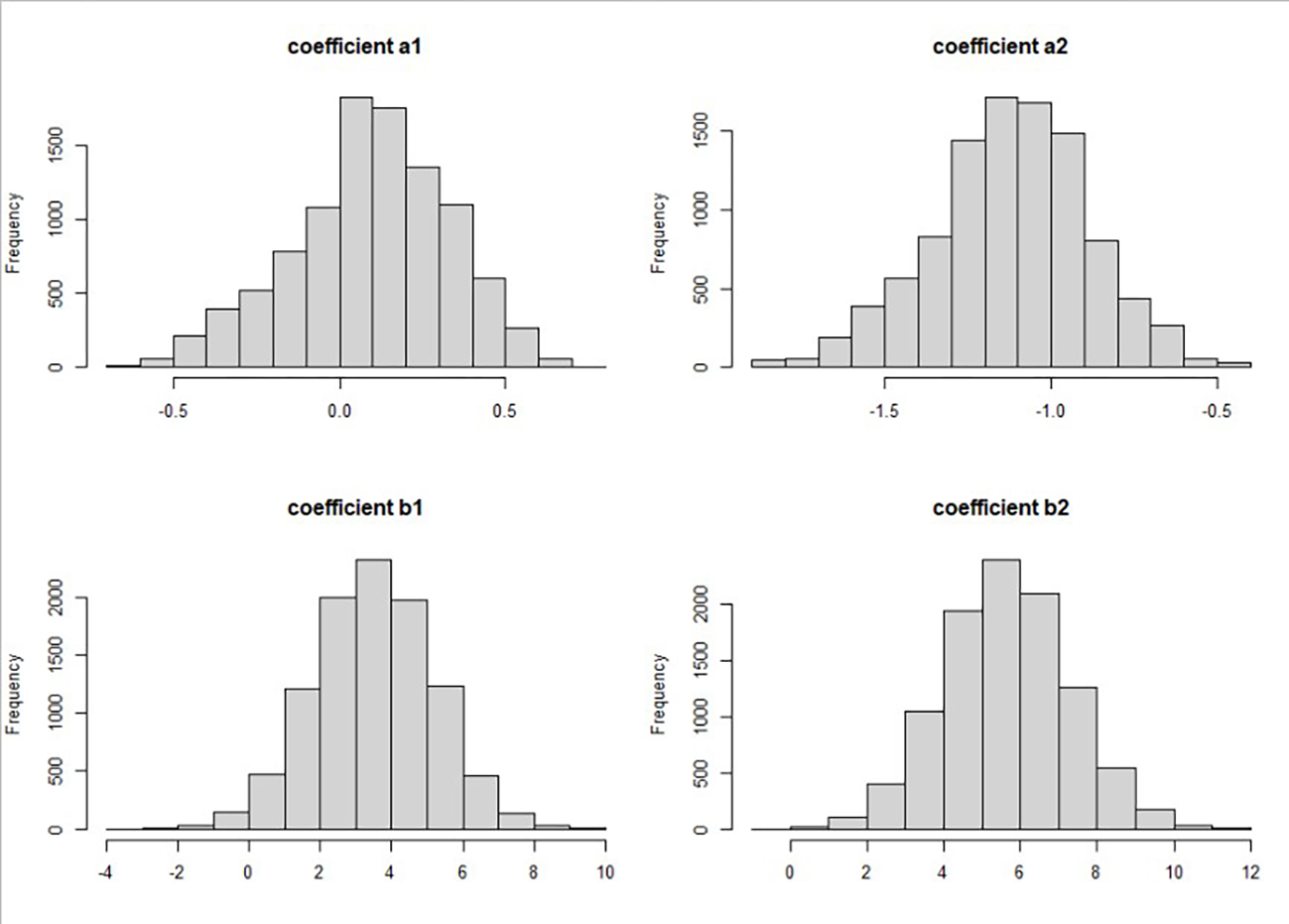
Posterior Posterior distribution for coefficients relating to the categorical variable insurance. The posterior distribution of the coefficient a1 for race to predict a log function of patients’ insurance status (**upper left panel**), the posterior distribution of the coefficient b1 for patients’ insurance status (public vs. no-insurance) to predict the diagnostic age of breast cancer (**lower left panel**), the posterior distribution of the coefficient a2 for race to predict a log function of patients’ insurance status (**upper right panel**), and the posterior distribution of the coefficient b2 for patients’ insurance status (private vs. no-insurance) to predict the diagnostic age of breast cancer (**lower right panel**).

Figure 4 shows the posterior distributions of the coefficients relating to insurance status. The posterior distribution of the coefficient $a_{1}$ is mostly likely to be positive, which means the odds of patients having public insurance over no insurance are likely to be higher among black women than among white women. The posterior distribution of the coefficient $a_{2}$ is negative, which means that the odds of patients having private insurance over no insurance are likely to be lower among black women than among white women. The posterior distribution of the coefficients for insurance indicators, $b_{1}$ and $b_{2}$, are both positive, which means that patients with private or public insurance were diagnosed at an older age on average than patients without insurance. The coefficient $b_{2}$ is larger than $b_{1}$, which means that patients with private insurance were diagnosed at an older age on average than those with public insurance. We included the posterior distributions for coefficients for other mediators and the R codes in the Supplementary Materials, which can be used to similarly explain the observed racial disparity.

## 5. Conclusions and Discussion

In summary, our paper investigated three Bayesian mediation methods: the function of coefficients method, the product of partial difference method, and the re-sampling method. Method 1 is most efficient when Bayesian generalized linear models are fitted. However, when it is difficult to derive functions of coefficients to estimate the mediation effects, especially for nonparametric models, Method 2 and Method 3 are more general and can be used with any predictive model. We applied these three methods to a CER-PCOR breast cancer data set to explore the racial disparity in the diagnostic age of breast cancer. The average diagnostic age of breast cancer for black women was significantly younger than that for white women. Using the Bayesian mediation analysis methods, we found that insurance and marital status can partially explain the disparity. However, the disparity cannot be fully explained after accounting for all variables in the study. There are variables for which data were not collected but that may influence the racial disparity, such as the type of cancer. As a next step, we will expand the variable collections. We will link the patients’ information with their hospital records to collect information on their breast cancer and comorbidities, and try to link the CER-PCOR breast cancer data set with social determinants of health variables to further explain the observed racial disparity. As a future study, we will also consider building multilevel models to account for risk factors at different levels. The implementation of multilevel models will be straightforward in the Bayesian setting.

## Supplementary Material

Supplementary Material

## Figures and Tables

**Table 1.** Summary statistics and test results for the identified mediators. *p*-value 1 gives the test results of whether the corresponding variable is significantly related to the outcome, adjusting for other variables. *p*-value 2 is the result of testing if the corresponding variable is distributed differently among AA and CA patients.

Potential Mediators	Black	White	*p*-Value 1	*p*-Value 2

Categorical Variables	Frequency (%)	Frequency (%)		

Insurance:			<0.0001	<0.0001
no insurance	3.74%	1.77%		
public insuance	34.63%	14.00%		
private insurance	61.64%	84.23%		

Marital status:			<0.0001	<0.0001
single	34.63%	11.97%		
married	35.49%	59.15%		
separated or divorced	14.51%	11.97%		
widowed	15.37%	16.91%		

Comorbidity:			<0.0001	<0.0001
no/mild	33.91%	40.53%		
moderate	59.20%	56.87%		
severe	6.90%	2.60%		

Stage:			<0.0001	0.0010
1. localized	53.74%	61.56%		
2. regional by direct extension only	1.58%	1.84%		
3. ipsilateral regional lymph nodes only	32.18%	26.54%		
4. regional by both 2 and 3	4.89%	3.10%		
5. distant sites	7.61%	6.97%		

Body mass index:			0.0030	0.0700
underweight	1.72%	2.15%		
healthy weight	14.80%	29.58%		
overweight	27.44%	30.59%		
obese	56.03%	37.68%		

**Continuous Variables**	**Mean (SD)**	**Mean (SD)**	***p*-Value 1**	***p*-Value 2**

Street density	15.29 (13.34)	9.87 (10.73)	0.3410	<0.0001
Intersection density	94.47 (105.26)	51.90 (73.45)	0.9180	<0.0001
Connected node ratio	0.71 (0.15)	0.63 (0.13)	0.0004	<0.0001
Language isolation	0.58 (0.15)	0.48 (0.17)	0.0270	<0.0001
Percent of persons under age 18	25.58 (5.50)	23.99 (5.01)	0.1000	<0.0001
Poverty index	25.29 (12.79)	15.04 (9.19)	<0.0001	<0.0001

**Table 2.** Summary of mediation effect estimations for racial/ethnic disparity in the diagnostic age of breast cancer.

Mediators	Effect (95% Credible Sets)	Relative Effect (%)

Insurance	−0.55 (−0.85, −0.26)	12.90 (6.05, 21.26)
Marital status	−0.74 (−1.20, −0.28)	17.17 (6.99, 28.22)
Comorbidity	0.12 (−0.05, 0.30)	−2.85 (−7.53, 1.23)
Stage	−0.14 (−0.26, −0.03)	3.27 (0.78, 6.33)
BMI	0.08 (−0.13, 0.28)	−1.81 (−7, 3.1)
Isolation	0.39 (0.04, 0.73)	−9.03 (−18.33, −1.11)
Poverty index	1.16 (0.65, 1.68)	−27.18 (−43.5, −14.53)
Under 18s	−0.13 (−0.30, 0.03)	3.07 (−0.63, 7.42)
J1 (walkability)	0.33 (0, 0.6642)	−7.79 (−16.28, −0.08)
Street density	0.04 (−0.76, 0.83)	−0.79 (−19.39, 18.45)
Intersection density	−0.40 (−1.21, 0.41)	9.02 (−10.21, 29.13)
Connected node ratio	0.68 (0.21, 1.16)	−16.02 (−28.85, −5.07)
Direct effect	−4.88 (−6.12, −3.65)	112.24 (94.02, 133.04)

Total effect	−4.37 (−5.47, −3.26)	

## Data Availability

To obtain the dataset, please contact Louisiana Tumor Registry.
