# Supplementary material for: Bayesian Mediation Analysis with an Application to Explore Racial Disparities in the Diagnostic Age of Breast Cancer
Source: Stats (Basel). Author manuscript; Available in PMC 2025 Jan 31. (PMC11784984; doi:10.3390/stats7020022)
Supplement: Supplementary Material [file NIHMS2008759-supplement-Supplementary_Material.pdf]

Supplemental Materials

1 CODES TO PERFORM BMA TO EXPLORE RACIAL DISPARITY IN BREAST CANCER DIAGNOSTIC AGE

```
M1=cbind(M1$Isolation,M1$poverty,M1$under18,M1$intden,
M1$StrDen,M1$cnr,M1$comorb1,M1$comorb2,M1$insurance1,
M1$insurance2,M1$marry1,M1$marry2,M1$marry3,M1$stage1,
M1$stage2,M1$stage3,M1$stage4,M1$BMI_c1,M1$BMI_c2,M1$BMI_c3)
############
contm=c(1,2,3,4,5,6)
p1=length(contm)
deltam=rep(0.01,p1)
contm1=matrix(data=c(1,2,3,4,5,6,1,2,3,4,5,6),6,2)
binm=NULL
p2=length(binm)
catm1=matrix(data=c(7,10,13,17,22,9,12,16,21,25),5,2)
catm=c(7,8,9,10,11)
p3=length(catm)
cat2=c(3,3,4,5,4)
cat1=max(cat2)-1
P=ncol(M1)
mu=rep(0,P)
Omega=diag(0.000001,P)
mu0.1=rep(0,P)
Omega0.1=diag(0.000001,P)
mu1.1=rep(0,P)
Omega1.1=diag(0.000001,P)
mu0.a=rep(0,p1)
```

```
Omega0.a=diag(0.000001,p1)
mu1.a=rep(0,p1)
Omega1.a=diag(0.000001,p1)
mu0.b=rep(0,p2)
Omega0.b=diag(0.000001,p2)
mu1.b=rep(0,p2)
Omega1.b=diag(0.000001,p2)
mu0.c=rep(0,cat1)
Omega0.c=diag(0.000001,cat1)
mu1.c=rep(0,cat1)
Omega1.c=diag(0.000001,cat1)
nrow(datanew0)
M22=M2[,c(3:8,15,10,11,12,14)]
nrow(M2)
data0<- list (N=2275,x=datanew0$race,y=datanew0$age_at_diagnosis,
M1=M1,M2=M22,contm=contm,contm1=contm1,p1=p1,
cat1=cat1,cat2=cat2,catm=catm,p3=p3,P=P,
mu=mu,Omega=Omega,mu0.1=mu0.1,mu1.1=mu1.1,Omega0.1=Omega0.1,
Omega1.1=Omega1.1,mu0.a=mu0.a,mu1.a=mu1.a,Omega0.a=Omega0.a,
Omega1.a=Omega1.a,mu0.c=mu0.c,mu1.c=mu1.c,Omega0.c=Omega0.c,
Omega1.c=Omega1.c)
inits<- function(){list()}
med0<- jags(data0, inits,
model.file = "C:/Users/wcao/Desktop/test1.txt",
parameters = c("beta0","c","beta","prec4","alpha0","alpha1","prec2",
"alpha0.a","alpha1.a","prec1","alpha0.c","alpha1.c"),
n.chains = 1, n.iter = 11000,n.burnin=1000,n.thin = 1)
#check the results
#calculate the mediation effects
N=2275
N1=10000
M3=M1
M3[,1:6]=M3[,1:6]+0.01
attach(med0$BUGSoutput$sims.list)
#method 1
aie1=matrix(0,N1,p1+p3)
for (j in 1: p1)
{aie1[,contm[j]]=alpha1.a[,j]*beta[,contm[j]]
```

```
}
for (j in 1:p3){
mu_Mc1<-matrix(0,N1,cat2[j]-1)
mu_Mc0<-matrix(0,N1,cat2[j]-1)
for (k in 2:cat2[j]){
mu_Mc1[,k-1] <- exp(alpha0.c[,j,k-1]+alpha1.c[,j,k-1])
mu_Mc0[,k-1] <- exp(alpha0.c[,j,k-1])
}
sum_Mc1 <-apply(mu_Mc1,1,sum)+1
sum_Mc0 <-apply(mu_Mc0,1,sum)+1
aie1[,catm[j]]<-diag((diag(1/sum_Mc1)%*%mu_Mc1-diag(1/sum_Mc0)%*%
mu_Mc0)%*%t(beta[,(catm1[j,1]-j+1):(catm1[j,2]-j)]))
}
de1=c
te1=apply(aie1,1,sum)+de1

#method2
aie2=matrix(0,N1,p1+p3)
ie2=array(0,dim=c(N1,N,p1))
for (j in 1:p1){
ie2[,,j]=(alpha1.a[,j]/deltam[j])*
(beta[,contm1[j,1]:contm1[j,2]]%*%t(M3[,contm1[j,1]:contm1[j,2]]
-M1[,contm1[j,1]:contm1[j,2]]))
aie2[,contm[j]]=apply(ie2[,,j],1,mean)
}

for (j in 1:p3){
mu_Mc1<-matrix(0,N1,cat2[j]-1)
mu_Mc0<-matrix(0,N1,cat2[j]-1)
for (k in 2:cat2[j]){
mu_Mc1[,k-1] <- exp(alpha0.c[,j,k-1]+alpha1.c[,j,k-1])
mu_Mc0[,k-1] <- exp(alpha0.c[,j,k-1])
}
sum_Mc1 <-apply(mu_Mc1,1,sum)+1
sum_Mc0 <-apply(mu_Mc0,1,sum)+1
aie2[,catm[j]]<-diag((diag(1/sum_Mc1)%*%mu_Mc1-diag(1/sum_Mc0)%*%
mu_Mc0)%*%t(beta[,(catm1[j,1]-j+1):(catm1[j,2]-j)]))
}
```

```
de2=c
te2=apply(aie2,1,sum)+de2

#method 3:parametric
ate3=NULL
aie3=NULL
for (m in 1:N1)
{mu_M2=M1
mu_M3=M1
for (j in 1:p1){
for (k in contm1[j,1]:contm1[j,2]){
mu_M2[,k] <- alpha0[m,k]+alpha1[m,k]
mu_M3[,k] <- alpha0[m,k]
}}

for (j in 1:p3){
mu_Mc2=rep(0,cat2[j]-1)
mu_Mc2[1:(cat2[j]-1)]<-exp(alpha0.c[m,j,(2:cat2[j])-1]+
alpha1.c[m,j,(2:cat2[j])-1])
sum_Mc2 <-sum(mu_Mc2)+1
mu_M2[,(catm1[j,1]-j+1):(catm1[j,2]-j)] <-
matrix(rep(mu_Mc2/sum_Mc2,each=N),N)
mu_Mc3=rep(0,cat2[j]-1)
mu_Mc3[1:(cat2[j]-1)] <- exp(alpha0.c[m,j,(2:cat2[j])-1])
sum_Mc3 <-sum(mu_Mc3)+1
mu_M3[,(catm1[j,1]-j+1):(catm1[j,2]-j)] <-
matrix(rep(mu_Mc3/sum_Mc3,each=N),N)
}

mu_y1<- rep(beta0[m,],each=N) + rep(c[m],each=N) + mu_M2%*%beta[m,]
mu_y0<- rep(beta0[m,],each=N) + mu_M3%*%beta[m,]
te3<- mu_y1-mu_y0
ate3=c(ate3,mean(te3))
j1=sample(1:N,replace=T)
j2=sample(1:N,replace=T)
ie=matrix(0,N,p1+p3)
#1. p1 is the total number of continuous mediators
for (j in 1:p1){
```

```
if(contm1[j,1]<contm1[j,2])
ie[,j]<-te3-(mu_y1-mu_M2[,contm1[j,1]:contm1[j,2]]%*%
beta[m,contm1[j,1]:contm1[j,2]]+
M1[j1,contm1[j,1]:contm1[j,2]]%*%beta[m,contm1[j,1]:contm1[j,2]]-
mu_y0+ mu_M3[,contm1[j,1]:contm1[j,2]]%*%
beta[m,contm1[j,1]:contm1[j,2]]-
M1[j1,contm1[j,1]:contm1[j,2]]%*%beta[m,contm1[j,1]:contm1[j,2]])
else
ie[,j]<-te3-(mu_y1-mu_M2[,contm1[j,1]]*beta[m,contm1[j,1]]+
M1[j1,contm1[j,1]]*beta[m,contm1[j,1]]-
mu_y0+ mu_M3[,contm1[j,1]]*beta[m,contm1[j,1]]-
M1[j1,contm1[j,1]]*beta[m,contm1[j,1]])
}
for (l in 1:p3){
ie[,l+p1+p2]<-te3-(mu_y1-mu_M2[,(catm1[l,1]-l+1):(catm1[l,2]-l)]
%*%beta[m,(catm1[l,1]-l+1):(catm1[l,2]-l)]+
M1[j1,(catm1[l,1]-l+1):(catm1[l,2]-l)]%*%
beta[m,(catm1[l,1]-l+1):(catm1[l,2]-l)]-
mu_y0+ mu_M3[,(catm1[l,1]-l+1):(catm1[l,2]-l)]%*%
beta[m,(catm1[l,1]-l+1):(catm1[l,2]-l)]-
M1[j1,(catm1[l,1]-l+1):(catm1[l,2]-l)]%*%
beta[m,(catm1[l,1]-l+1):(catm1[l,2]-l)])
}
aie3=rbind(aie3,apply(ie,2,mean))
}
de3=c


#method 3: nonparametric
M111=cbind(M1,datanew0[,1])
M.0=M111[M111[,21]==1,]
M.1=M111[M111[,21]==2,]
M.0=M.0[,-21]
M.1=M.1[,-21]
ate4=NULL
aie4=NULL

for(m in 1:N1){
```

```
j1=sample(1:N,replace = T)
j2=sample(1:N,replace = T)
j3=sample(1:nrow(M.0),N,replace = T)
j4=sample(1:nrow(M.1),N,replace = T)
te<- c[m] + (M.1[j4,]-M.0[j3,])%*%beta[m,]
ate4=c(ate4,mean(te))
#ie for continuous mediators
ie=matrix(0,N,p1+p2+p3)
for (j in 1:p1){
M.1.0=M.1[j4,]
M.0.0=M.0[j3,]
M.1.0[,contm1[j,1]:contm1[j,2]]=M1[j1,contm1[j,1]:contm1[j,2]]
M.0.0[,contm1[j,1]:contm1[j,2]]=M1[j2,contm1[j,1]:contm1[j,2]]
ie[,contm[j]]<-te-c[m]-(M.1.0-M.0.0)%*%beta[m,]
}
#ie for categorical mediators
for (j in 1:p3){
M.1.0=M.1[j4,]
M.0.0=M.0[j3,]
M.1.0[,(catm1[j,1]-j+1):(catm1[j,2]-j)]=
M1[j1,(catm1[j,1]-j+1):(catm1[j,2]-j)]
M.0.0[,(catm1[j,1]-j+1):(catm1[j,2]-j)]=
M1[j1,(catm1[j,1]-j+1):(catm1[j,2]-j)]
ie[,catm[j]]<-te-c[m]-(M.1.0-M.0.0)%*%beta[m,]
}
aie4=rbind(aie4,apply(ie,2,mean))
}
de4<-c
summary.med<-function(vec,qua=c(0.025, 0.25, 0.5, 0.75,0.975), digit=4)
{round(c(mean=mean(vec),sd=sd(vec),quantile(vec,qua)),digit)}
summary.med.re<-function(vec,vec1,
qua=c(0.025, 0.25, 0.5, 0.75,0.975),digit=4)
{vec=vec/vec1
round(c(mean=mean(vec),sd=sd(vec),quantile(vec,qua)),digit)}
result1<-apply(cbind(te1,de1,aie1),2,summary.med)
result4<-apply(cbind(ate4,de4,aie4),2,summary.med)
colnames(result1)=c("TE","DE","Isolation", "poverty","under18",
"intden", "StrDen", "cnr","comorb",
```

```
"insurance", "marstat1","Satge","BMI")
colnames(result4)=c("TE","DE","Isolation", "poverty","under18",
"intden", "StrDen", "cnr", "comorb",
"insurance", "marstat1","Satge","BMI")
result1
result4
```

2 JAGS MODEL FOR BMA TO EXPLORE RACIAL DISPARITY IN BREAST CANCER DIAGNOSTIC AGE

```
model {
for(i in 1:N){
mu_y[i]<- beta0 + c*x[i] + inprod(beta,M1[i,])
y[i] ~ dnorm(mu_y[i],prec4)
for (j in 1:p1){
mu_M1[i,j] <- alpha0.a[j]+alpha1.a[j]*x[i]
M2[i,contm[j]] ~ dnorm(mu_M1[i,j],prec1[j])
for (k in contm1[j,1]:contm1[j,2]){
mu_M1_c[i,k] <- alpha0[k]+alpha1[k]*x[i]
M1[i,k] ~ dnorm(mu_M1_c[i,k],prec2[k])
}
}

for (j in 1:p3){
mu_Mc[i,j,1] <- 1 #baseline is the 1st category
for (k in 2:cat2[j]){
mu_Mc[i,j,k] <- exp(alpha0.c[j,k-1]+alpha1.c[j,k-1]*x[i])
}
sum_Mc[i,j] <-sum(mu_Mc[i,j,1:cat2[j]])
for (l in 1:cat2[j])
{mu_Mc0[i,j,l] <- mu_Mc[i,j,l]/sum_Mc[i,j]}
M2[i,catm[j]]~dcat(mu_Mc0[i,j,1:cat2[j]])

}
}
```

```
#priors
beta[1:P] ~ dmnorm(mu[1:P], Omega[1:P, 1:P])
alpha0[1:P] ~ dmnorm(mu0.1[1:P], Omega0.1[1:P, 1:P])
alpha1[1:P] ~ dmnorm(mu1.1[1:P], Omega1.1[1:P, 1:P])
for (i in 1:p1){
var2[i] ~ dgamma(1,0.1)
prec2[i] <-1/var2[i]
}
#3. alpha0.a, alpha1.a, prec1
alpha0.a[1:p1] ~ dmnorm(mu0.a[1:p1], Omega0.a[1:p1, 1:p1])
alpha1.a[1:p1] ~ dmnorm(mu1.a[1:p1], Omega1.a[1:p1, 1:p1])
for (i in 1:p1){
var1[i] ~ dgamma(1,0.1)
prec1[i] <-1/var1[i]
}
#5. alpha0.c, alpha1.c
#cat1=max(cat2)-1
for (i in 1:p3){
alpha0.c[i,1:cat1] ~ dmnorm(mu0.c[1:cat1], Omega0.c[1:cat1, 1:cat1])
alpha1.c[i,1:cat1] ~ dmnorm(mu1.c[1:cat1], Omega1.c[1:cat1, 1:cat1])
}
beta0 ~ dnorm(0, 1.0E-6)
c ~ dnorm(0, 1.0E-6)
var4 ~ dgamma(1,0.1)
prec4 <-1/var4
}
```

3 Figures

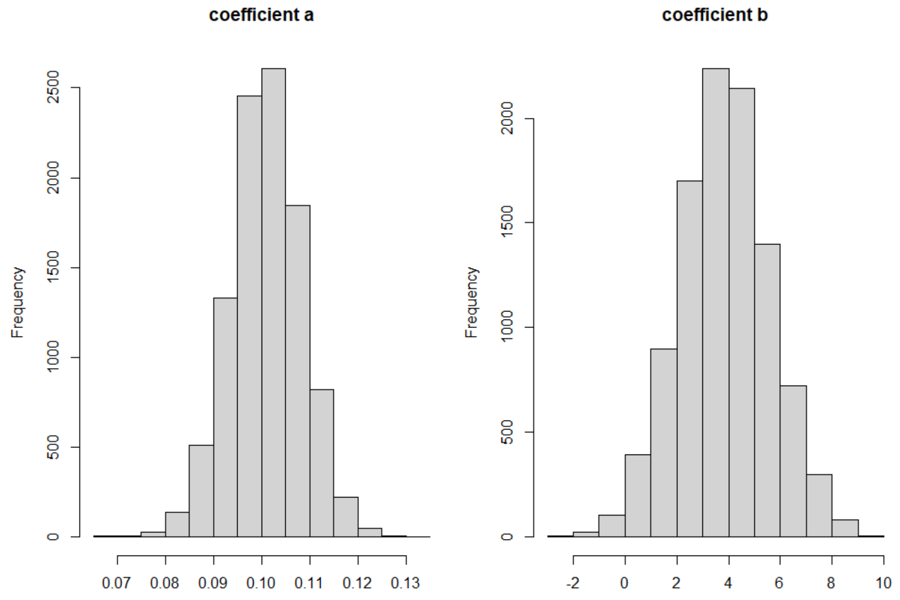

Figure 1: Posterior distribution for coefficients relating to language isolation. The posterior distribution of the coefficient a for race to predict language isolation (left panel), and the posterior distribution of the coefficient b for language isolation to predict the diagnostic age of breast cancer (right panel).

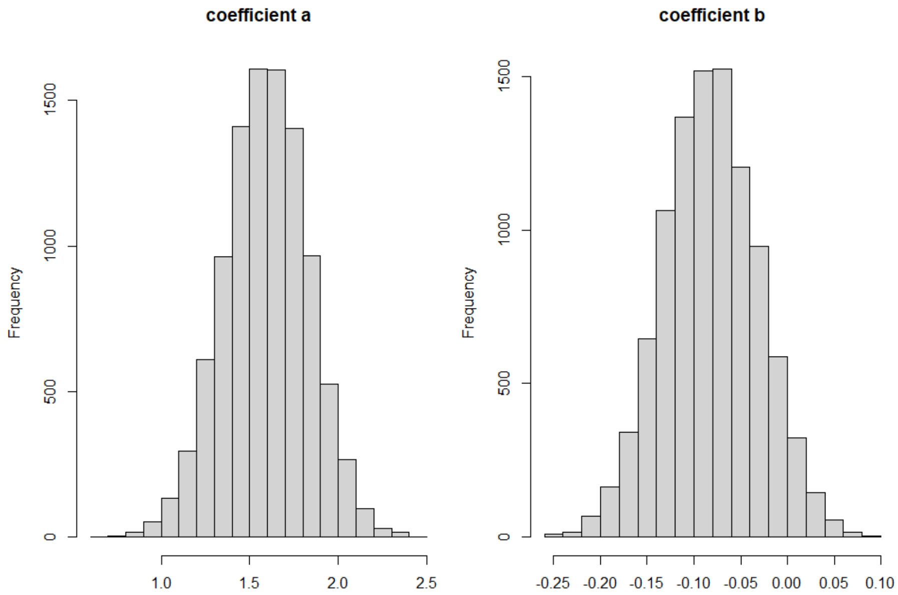

Figure 2: Posterior distribution for coefficients relating to percentage of persons under age 18. The posterior distribution of the coefficient a for race to predict percentage of persons under age 18 (left panel), and the posterior distribution of the coefficient b for percentage of persons under age 18 to predict the diagnostic age of breast cancer (right panel).

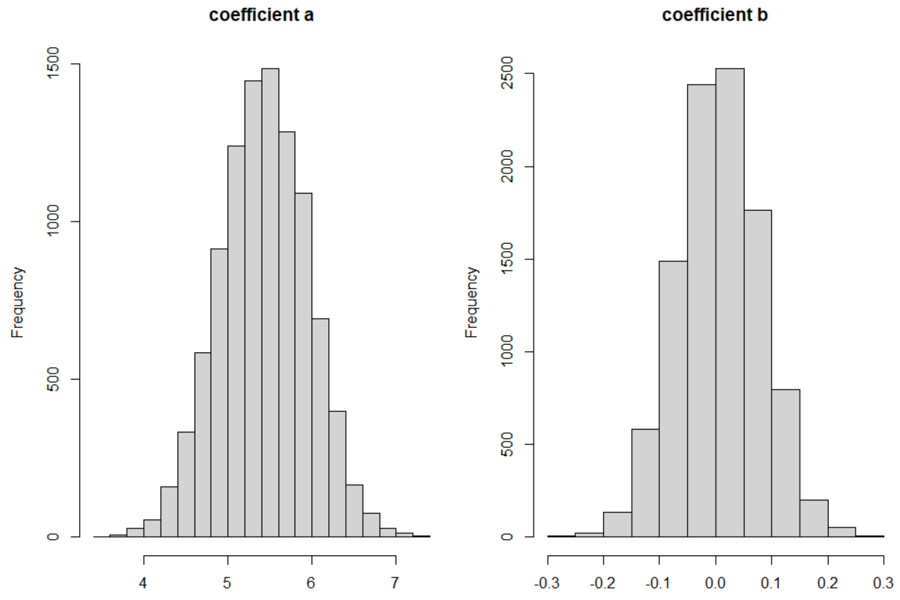

Figure 3: Posterior distribution for coefficients relating to street density. The posterior distribution of the coefficient a for race to predict street density (left panel), and the posterior distribution of the coefficient b for street density to predict the diagnostic age of breast cancer (right panel).

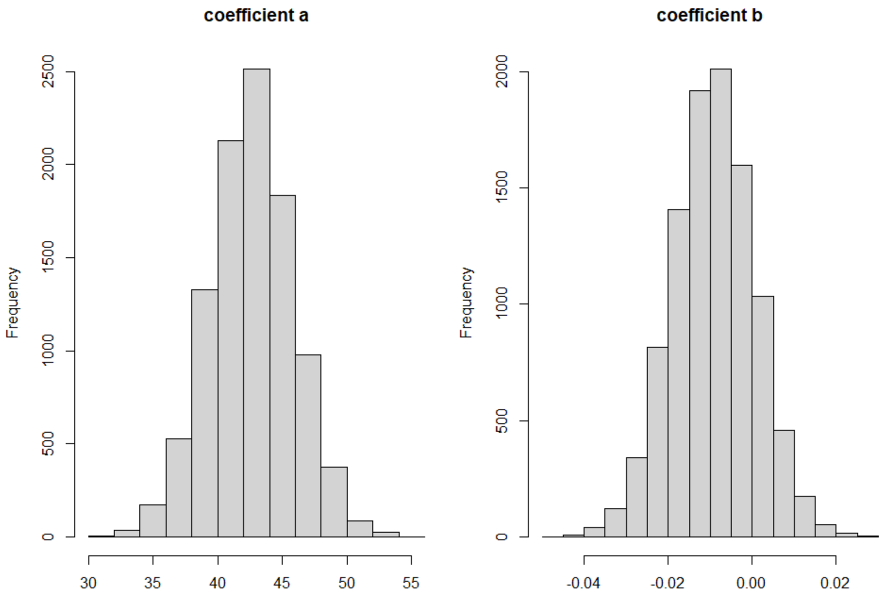

Figure 4: Posterior distribution for coefficients relating to intersection density. The posterior distribution of the coefficient a for race to predict intersection density (left panel), and the posterior distribution of the coefficient b for intersection density to predict the diagnostic age of breast cancer (right panel).

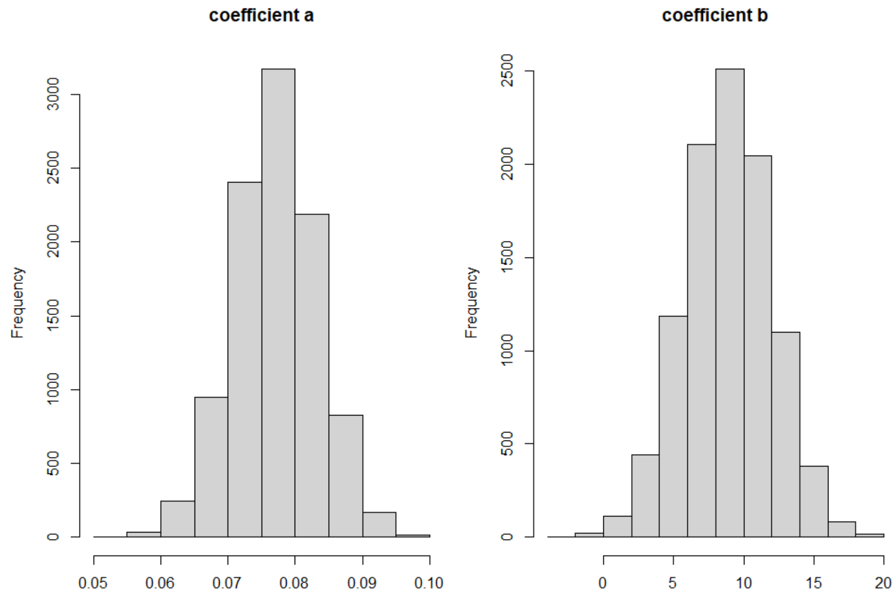

Figure 5: Posterior distribution for coefficients relating to street connected node ratio. The posterior distribution of the coefficient a for race to predict street connected node ratio (left panel), and the posterior distribution of the coefficient b for street connected node ratio to predict the diagnostic age of breast cancer (right panel).

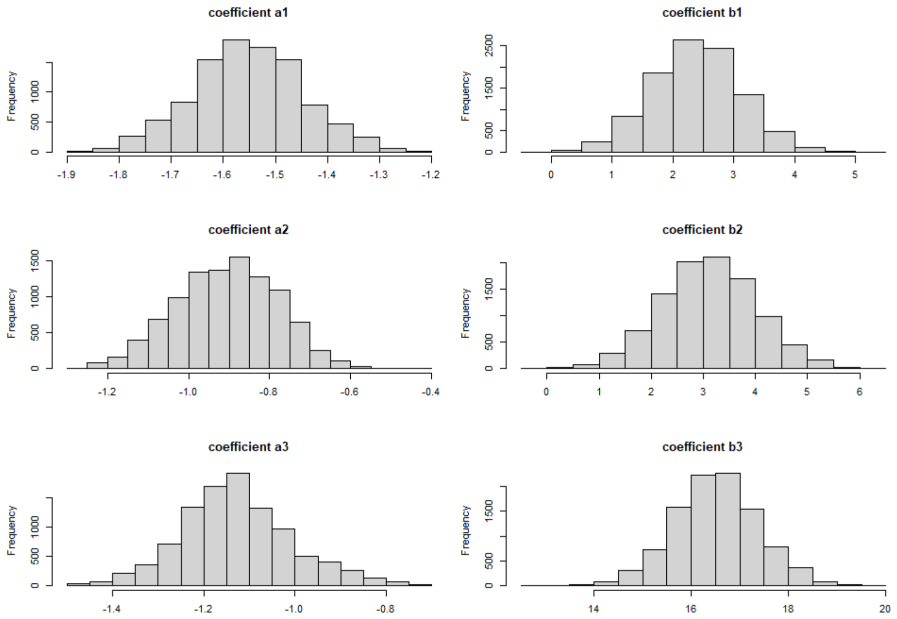

Figure 6: Posterior distribution for coefficients relating to the categorical variable-marital status. The posterior distribution of the coefficient a1, a2, a3 for race to predict a log function of patients' marital status (left panel), the posterior distribution of the coefficient b1 for patients' marital status (married vs single) to predict the diagnostic age of breast cancer (upper right panel), the posterior distribution of the coefficient b2 for patients' marital status (separated/divorce vs single) to predict the diagnostic age of breast cancer (middle right panel), and the posterior distribution of the coefficient b3 for patients' marital status (widowed vs single) to predict the diagnostic age of breast cancer (lower right panel).

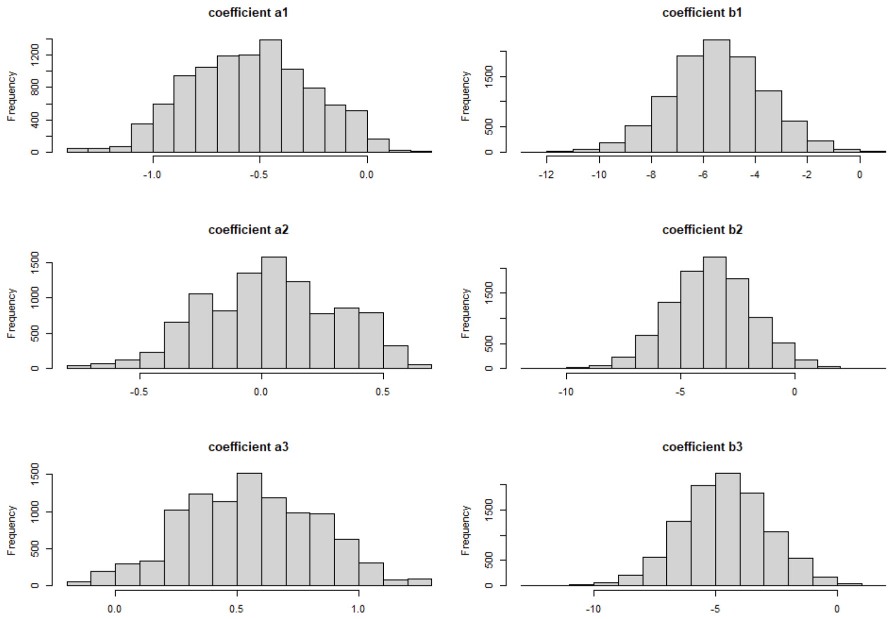

Figure 7: Posterior distribution for coefficients relating to the categorical variable-BMI. The posterior distribution of the coefficient a1, a2, a3 for race to predict a log function of patients' BMI (left panel), the posterior distribution of the coefficient b1 for patients' BMI (healthy weight vs underweight) to predict the diagnostic age of breast cancer (upper right panel), the posterior distribution of the coefficient b2 for patients' BMI (overweight vs underweight) to predict the diagnostic age of breast cancer (middle right panel), and the posterior distribution of the coefficient b3 for patients' BMI (obesity vs underweight) to predict the diagnostic age of breast cancer (lower right panel).

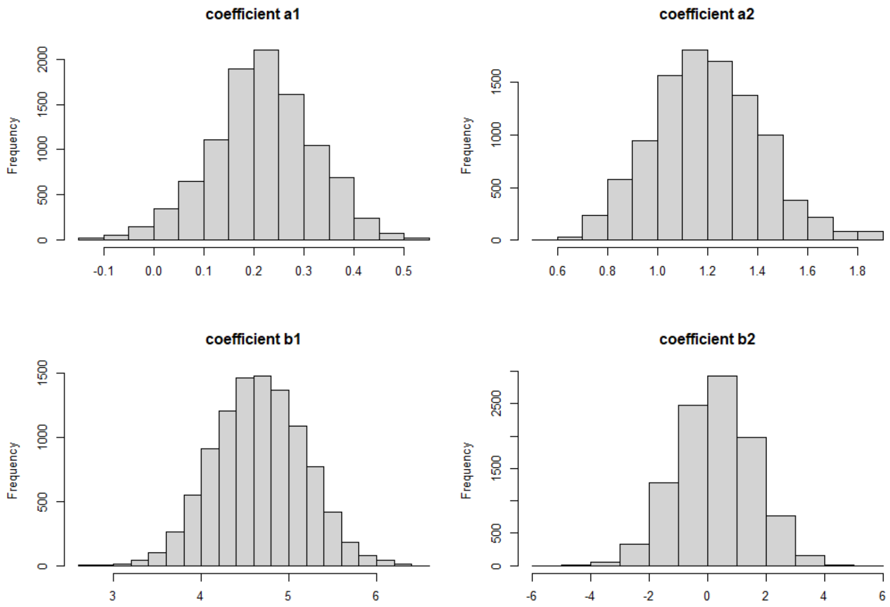

Figure 8: Posterior distribution for coefficients relating to the categorical variable-comorbidity. The posterior distribution of the coefficient a1, a2, a3 for race to predict a log function of patients' comorbidity (left panel), the posterior distribution of the coefficient b1 for patients' comorbidity (moderate vs no/mild) to predict the diagnostic age of breast cancer (upper right panel),and the posterior distribution of the coefficient b2 for patients' comorbidity (severe vs no/mild) to predict the diagnostic age of breast cancer (lower right panel)

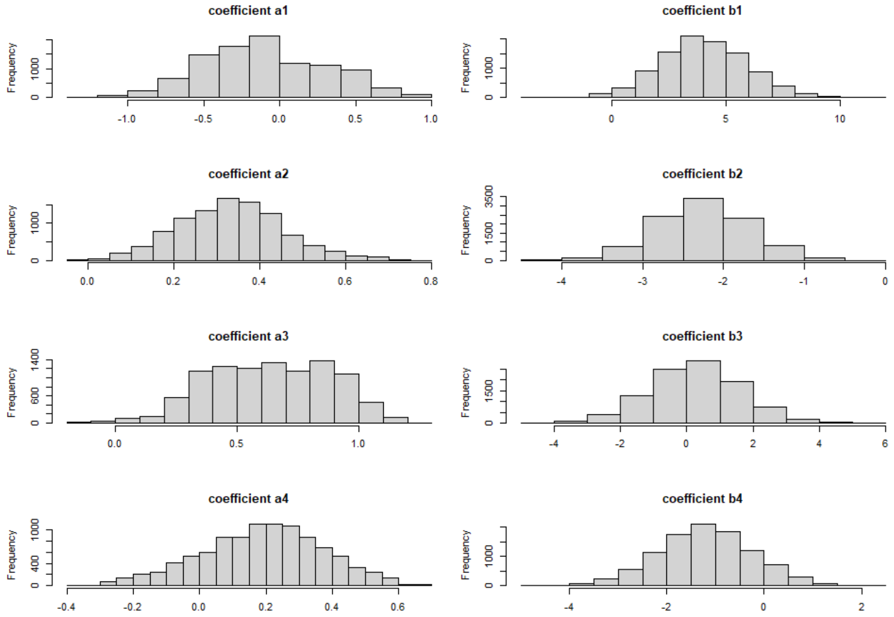

Figure 9: Posterior distribution for coefficients relating to the categorical variable-cancer stage. The posterior distribution of the coefficient a1, a2, a3, a4 for race to predict a log function of patients' cancer stage (left panel), the posterior distribution of the coefficient b1 for patients' cancer stage (regional by direct extension only vs localized) to predict the diagnostic age of breast cancer (upper right panel), the posterior distribution of the coefficient b2 for patients' cancer stage (ipsilateral regional lympg nodes only vs localized) to predict the diagnostic age of breast cancer (middle right panel), the posterior distribution of the coefficient b3 for patients' cancer stage (regional by 2 and 3 vs localized) to predict the diagnostic age of breast cancer (middle right panel),and the posterior distribution of the coefficient b4 for patients' cancer stage (distant sites vs localized) to predict the diagnostic age of breast cancer (lower right panel).
